# Complete Response to Pembrolizumab in Stage IV Alveolar Soft Part Sarcoma After Failure of Four Lines of Treatment: A Case Report and Literature Review

**DOI:** 10.7759/cureus.62094

**Published:** 2024-06-10

**Authors:** Parisa Aijaz, Hassan Sohail, Muhammad A Niazi, Amir Kamran

**Affiliations:** 1 Internal Medicine, Charleston Area Medical Center, Charleston, USA; 2 Internal Medicine, Dow University of Health Sciences, Karachi, PAK; 3 Hematology-Oncology, Charleston Area Medical Center, Charleston, USA

**Keywords:** sarcoma, oncology, case report, pembrolizumab, alveolar soft-part sarcoma

## Abstract

Alveolar soft part sarcoma (ASPS) is a rare malignant tumor that manifests as a slow-growing soft tissue mass and frequently presents with distant metastasis. The prognosis is variable, and complete remission of metastatic disease has rarely been reported. Our patient was diagnosed with metastatic ASPS at the age of 17, with a primary forearm lesion and metastasis to the lungs. She underwent surgical resection of her forearm mass, followed by adjuvant chemotherapy and radiation to target the lung metastasis. Over the next decade, she had a complicated course of treatment. Her disease continued to slowly progress despite treatment with sunitinib, pazopanib, and a combination of docetaxel and gemcitabine. We eventually treated her with immune checkpoint inhibitors (ICIs). Pembrolizumab, initially in combination with bevacizumab and later as monotherapy, resulted in significant tumor shrinkage, especially in the pulmonary lesions, within the first three months. Subsequent imaging reported complete remission within 15 months and no disease recurrence at her three-year follow-up. Our case highlights one of the very few reported cases of complete remission achieved in metastatic ASPS after treatment with ICIs. ICIs could offer hope for disease remission in advanced ASPS, a rare malignancy that has proven difficult to treat successfully in the past. More studies need to be conducted to further evaluate the efficacy and any associated predictors of successful treatment.

## Introduction

Alveolar soft part sarcoma (ASPS) is an uncommon tumor that often appears in the soft tissues of the head and neck area in children and the lower limbs in adults. It usually manifests as a slowly expanding mass or as an illness that has already been disseminated. It makes up 0.2-0.9% of all soft tissue sarcomas [1], usually manifests in people between the ages of 15 and 35, and is uncommon in those under the age of five or above the age of 50 [2]. Despite having slow-growing features, 20-40% of ASPS present with metastasis [3], with the lungs being the most common site of metastasis. We present the case of a young female with ASPS and lung metastasis, wherein successful complete remission was achieved with immune checkpoint inhibitors (ICIs).

## Case presentation

**Figure 1:**
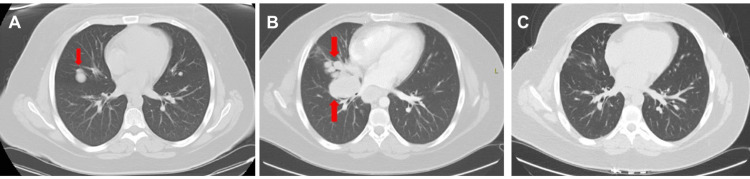
CT of the chest showing (A) initial lung nodules in 2011, (B) progression of the lung nodules in 2015, and (C) complete remission of the lung nodules in 2023

This report details the clinical course (Table 1) of a 17-year-old female who presented with a slow-growing mass in her left forearm for the past two years. The mass was surgically resected, and genetic analysis of the resected tissue revealed a translocation X;17 (P11.2: 225), confirming the diagnosis of ASPS. A CT scan of the chest reported lung nodules (Figure 1A), and it was determined that she has stage IV metastatic ASPS. Adjuvant chemotherapy and radiation were administered, resulting in remission of the lung nodules. Three years later, in 2014, the recurrence of the lung mass prompted the initiation of sunitinib. Despite treatment with sunitinib, a 2.4 × 1.9 cm brain lesion was identified on an MRI of the brain in the right parietal lobe a year later. This required a complete surgical resection of the brain mass and switching therapy to pazopanib. Her disease continued to progress in the form of pulmonary (Figure 1B) and hepatic lesions. She received palliative brain radiation, and pazopanib was discontinued. The disease was stable based on a surveillance PET/CT scan and brain MRI in November 2015. Progression was again observed on a PET/CT scan and brain MRI in June 2017. Palliative brain radiation and pazopanib were reintroduced; however, the lung masses continued to slowly enlarge and increase, necessitating switching therapy to docetaxel and gemcitabine in June 2019. Continued disease progression led to the discontinuation of this regimen after five cycles. Pembrolizumab, initially in combination with bevacizumab, then as monotherapy, offered a turning point in November 2019. Within three months, tumor shrinkage was observed. The most remarkable response was the complete resolution of pulmonary lesions (Figure 1C). By March 2021, complete remission was achieved with no evidence of solid masses or lymphadenopathy on six monthly scans. Pembrolizumab administration continues as a maintenance therapy every four weeks. Her most recent brain MRI and CT chest, abdomen, and pelvis were in June 2023, without evidence of disease recurrence.

**Table 1:** Detailed clinical course of a patient who had complete remission of stage IV ASPS with pembrolizumab treatment ASPS, alveolar soft part sarcoma

Timeline	Imaging findings	Intervention	Outcome
2010 (initial diagnosis)	Forearm mass + pulmonary metastasis	Surgical resection of forearm mass + adjuvant chemotherapy and radiation	Disease stable for three years
2014	Recurrence of pulmonary masses	Initiated sunitinib	Disease progression for one year
5/2015	2.4 × 1.9 cm right parietal lobe brain mass	Tumor resection + brain irradiation; initiated pazopanib	Disease stable for two years
6/2017	Progression of lung masses in the pleural bases, right hilum, and carina	Continued pazopanib	Disease progression for two years
6/2019	Continued progression of pulmonary masses and lymphadenopathy	Switched therapy to docetaxel + gemcitabine; received five cycles	Disease progression for five months
11/2019	New lymphadenopathy in the jugular chain	Switched therapy to pembrolizumab + bevacizumab for three cycles	Disease regression
2/2020	Right lung mass shrunk to 2.3 cm from 4.2 cm; right hilar mass shrunk to 3.5 cm from 6.1 cm; complete resolution of smaller pulmonary masses	Continued pembrolizumab q4 weeks	Continued disease regression
7/2020	Pulmonary masses continued to decrease in size on PET/CT; no new masses on the brain MRI	Continued pembrolizumab q4 weeks	Continued disease regression
12/2020	No new nodules or lymphadenopathy	Continued pembrolizumab q4 weeks	Continued disease regression
3/2021	No evidence of pulmonary, hepatic, or brain masses; no evidence of lymphadenopathy	Continued pembrolizumab q4 weeks.	Complete disease remission
1/2022	No evidence of new masses	Continued pembrolizumab q4 weeks	Complete disease remission; no disease recurrence
6/2023	No evidence of new masses	Continued pembrolizumab q4 weeks	Complete disease remission; no disease recurrence

## Discussion

ASPS is an uncommon tumor that appears in the soft tissues of the head and neck area in children and the lower limbs in adults and was first described by Christopherson et al. in 1952 [4]. It usually manifests as a slowly expanding mass or as an illness that has already been disseminated. It makes up 0.2-0.9% of all soft tissue sarcomas [1]. ASPS usually manifests in people between the ages of 15 and 35 and is uncommon in those under the age of five or above the age of 50 [2]. In a 2:1 ratio, it affects females more frequently than males. This ratio is more prevalent in the first three decades of life and then exhibits a little masculine predominance in later years [5]. Adult cases of ASPS typically affect the buttocks and thighs’ deep soft tissues. The tongue and orbital areas are the most often affected areas of the head and neck in children and newborns with ASPS [2].

Recent research has revealed that the der(17)t(X;17) (p11; q25) of human ASPS fuses the ASPL gene at 17q25 with the transcription factor 3 (TFE3) gene at Xp11. This results in an ASPL-TFE3 fusion protein, which causes deregulation of transcription in ASPS [6]. More recently, a very sensitive and specific marker for ASPS has been identified: an antibody against TFE3’s C-terminus [7]. While TFE3 expression appears to be practically ubiquitous in normal tissues, it is expressed at extremely low levels, and tumors known to have TFE3 gene fusions, such as ASPSs and uncommon pediatric renal carcinomas, are the only places where high nuclear expression of TFE3 is observed [7].

Despite having slow-growing features, 20-40% of ASPS present with metastasis [8], with the lungs being the most common site of metastasis. Consistent with this, our patient had lung metastasis on presentation. The next most common sites of metastasis are the brain and bones [9]. Our patient presented with lung metastasis on presentation.

According to one study, the incidence of ASPS-related brain metastases was estimated to be 30%, and the median survival time following a diagnosis of brain metastases was short: 12 months [10]. In an additional research project by Lin et al. [11], nine patients (69.2%) had lung metastasis; two of these patients additionally experienced brain and bone metastases (7.7% and 7.7%, respectively).

Multiple imaging modalities can be utilized to evaluate ASPS, and the diagnosis is confirmed via biopsy and histopathological evaluation. The plane view may show soft tissue calcification from a radiological perspective. On contrast-enhanced CT or angiography, ASPS presents as hypervascular lesions with convoluted, dilated veins and an intense tumor stain [12]. On T1- and T2-weighted images, MRI usually displays a high signal intensity; contrast-enhanced MRI, on the other hand, shows many peritumoral and intratumoral tortuous signal voids as well as significant enhancement [12,13].

The best course of treatment for ASPS primary and metastatic tumors is surgical resection; however, choosing the right adjuvant regimen proves to be difficult. Recent studies have suggested that radiation therapy may prevent or lessen local recurrences.

Eight instances of orbital ASPS were described by Hei et al. [13]; six of those patients had postoperative radiation treatment, and five of those cases showed a good prognosis with no signs of metastasis or local recurrence over the follow-up period (which ranged from three to 61 months). Gamma knife radiation treatment or stereotactic body radiation therapy might provide adequate local control for primary intracranial ASPS or brain metastases of ASPS; the median progression-free survival was reached 12 months following gamma knife radiation [10].

Chemotherapy’s effectiveness on ASPS has not yet been adequately proven. Retrospective analysis of the first-line treatment response in 68 ASPS patients by Reichardt et al. [14] revealed that 51% of patients had progressed disease, 41% had stable disease (SD), and just 4% had a complete response.

Multitargeted tyrosine kinase inhibitors have been shown in an increasing number of targeted treatment trials for ASPS to be effective [15]. The National Comprehensive Cancer Network (NCCN) and the Chinese Society of Clinical Oncology (CSCO) recommend sunitinib and pazopanib for the treatment of ASPS. According to reports, sunitinib has potential effectiveness for ASPS [16]. When sunitinib was given to patients with metastatic ASPS in a retrospective investigation, the median patient-free survival was 17 months. In our case, in 2014, following main surgery and adjuvant chemoradiation treatment, sunitinib was initiated due to a recurrence of the lung tumor. A year later, a 2.4 × 1.9 cm brain lesion in the right parietal lobe was discovered, despite receiving sunitinib therapy.

It has also been observed that pazopanib, a different tyrosine kinase inhibitor, works well against vascular endothelial growth factor in the treatment of ASPS [17]. In our patient, the brain lesion was completely surgically removed before pazopanib was begun, but then there was more disease development, resulting in hepatic and lung metastasis, which led to brain radiation and the discontinuation of pazopanib. Even reintroducing pazopanib had no beneficial effect, and the lung masses continued to slowly enlarge and increase, necessitating switching therapy to docetaxel and gemcitabine.

ICIs are being used more often in late-stage patients with malignant tumors that have not responded to multiple treatments, such as melanoma, renal cell carcinoma, and non-small-cell lung cancer, and they have produced a higher antitumor response [18]. ICIs include anti-programmed death-1 (PD-1), anti-PD-L1, and anti-cytotoxic T lymphocyte antigen 4. Two cases of ASPS were described by Kuo et al. [19]. One patient, who had failed therapy with various target medications, was given nivolumab (a PD-1 inhibitor), and the SD was maintained for seven months. Guidelines from the NCCN and CSCO have only suggested pembrolizumab as a therapy for ASPS thus far. In this case, pembrolizumab promised a paradigm shift in November 2019, first in conjunction with bevacizumab and later as monotherapy. A substantial tumor reduction was seen after three months. Complete clearance of pulmonary lesions was the most notable result. A retrospective analysis of the charts of 50 consecutive patients with metastatic or incurable advanced sarcomas supported the advice for pembrolizumab, and checkpoint inhibitors were found to be clinically beneficial for all four ASPS patients [20].

## Conclusions

This case presents a rare instance of long-term disease remission in advanced ASPS achieved with pembrolizumab. While targeted therapies are increasingly explored in sarcomas, their efficacy in ASPS remains under investigation. We highlight the challenging nature of ASPS and the potential of utilizing ICIs, specifically pembrolizumab, to successfully treat the disease.
